# Neurodevelopment assessment of small for gestational age children in a community-based cohort from Pakistan

**DOI:** 10.1136/archdischild-2022-324630

**Published:** 2022-10-26

**Authors:** Sabahat Naz, Zahra Hoodbhoy, Ali Jaffar, Sidra Kaleem, Babar S Hasan, Devyani Chowdhury, Melissa Gladstone

**Affiliations:** 1 Department of Pediatrics and Child Health, The Aga Khan University, Karachi, Sindh, Pakistan; 2 Cardiology Care for Children, Philadelphia, Pennsylvania, USA; 3 Department of Women and Children's Health, University of Liverpool, Liverpool, UK

**Keywords:** child development, paediatrics, child health

## Abstract

**Background:**

Children born small for gestational age (SGA) may experience more long-term neurodevelopmental issues than those born appropriate for gestational age (AGA). This study aimed to assess differences in the neurodevelopment of children born SGA or AGA within a periurban community in Pakistan.

**Methods:**

This was a prospective cohort study in which study participants were followed from the pilot Doppler cohort study conducted in 2018. This pilot study aimed to develop a pregnancy risk stratification model using machine learning on fetal Dopplers. This project identified 119 newborns who were born SGA (2.4±0.4 kg) based on International Fetal and Newborn Growth Consortium standards. We assessed 180 children (90 SGA and 90 AGA) between 2 and 4 years of age (76% of follow-up rate) using the Malawi Developmental Assessment Tool (MDAT).

**Findings:**

Multivariable linear regression analysis comparing the absolute scores of MDAT showed significantly lower fine motor scores (β: −0.98; 95% CI −1.90 to –0.06) among SGAs, whereas comparing the z-scores using multivariable logistic regression, SGA children had three times higher odds of overall z-scores ≤−2 (OR: 3.78; 95% CI 1.20 to 11.89) as compared with AGA children.

**Interpretation:**

SGA exposure is associated with poor performance on overall MDAT, mainly due to changes in the fine motor domain in young children. The scores on the other domains (gross motor, language and social) were also lower among SGAs; however, none of these reached statistical significance. There is a need to design follow-up studies to assess the impact of SGA on child’s neurodevelopmental trajectory and school performance.

**WHAT IS ALREADY KNOWN ON THIS TOPIC**

- Neurodevelopment issues are common among children under the age of 5 years, with the highest number of children (250 million) reported from low-income and middle-income countries (LMICs).
- These children are unable to meet their developmental milestones, putting them at risk of poor school performance and limited work and learning opportunities later in life.
- There is evidence regarding children born small for gestational age (SGA) as a risk factor for neurodevelopmental delays in high-income countries. However, there is limited literature available, from LMICs where the burden of SGA and neurodevelopmental delays is high.

**WHAT THIS STUDY ADDS**

- Using the Malawi Development Assessment Tool (MDAT), which is culturally appropriate, validated and explicitly design for LMICs, this study provides evidence of significant differences in the MDAT overall z-scores and fine motor absolute scores among SGA children as compared with AGA from a community-based cohort in Karachi, Pakistan.

**HOW THIS STUDY MIGHT AFFECT RESEARCH, PRACTICE OR POLICY**

- This study found a higher number of SGA children with overall neurodevelopmental deficits, specifically due to the fine motor domain, which in the MDAT is closely linked to cognitive abilities and are known to correlate with a child’s current and future school performance.
- The findings provide the grounds for screening of neurodevelopmental issues using MDAT among children born SGA in LMICs. Such children need referral to higher levels of care for further management.

## Introduction

Early child development (ECD) is the foundation for childrens’ overall health and well-being and reflects their future capabilities and productivity.^1^ Early childhood is a period during which children are exposed to different environmental, lifestyle and psychosocial circumstances that promote their physiological and neurodevelopmental growth.^2 3^ Those with inadequate ECD opportunities are at greater risk of having poor school performance and compromised work and earning opportunities later in life.^4^ Therefore, ECD is important to empower children to thrive and improve their learning outcomes, ultimately increasing opportunities and their productivity in the future.^5^

Globally, neurodevelopment delays are a major long-term issue among children under 5 years, with no significant change in the prevalence of neurodevelopmental problems in 2016 (8.4%, 95% CI 7.7 to 9.1) as compared with 1990 (8.9%, 95% CI 8.2 to 9.5).^6 7^ Furthermore, in low-income and middle-income countries (LMICs), it is estimated that around 250 million children under 5 years do not reach their developmental potential.^8^ The literature from South Asian countries has reported a high frequency of developmental delays (33%) using a combined indicator of early child development index in under-five children.^9^ Risk factors associated with these issues include low socioeconomic status, lack of parental education, inadequate nutrition, poor healthcare services and adverse in utero environments leading to children born small for gestational age (SGA).^10 11^ SGA is a condition in which the fetus fails to grow to its biological potential due to various factors, including placental insufficiency and intrauterine infections, leading to short-term and long-term issues later in life, including cardiovascular, immunological and neurodevelopment disorders.^12 13^

Children born SGA have higher odds of developing neurodevelopment issues, including motor delay, cognitive disability and memory issues, than those born appropriate for gestational age (AGA).^14^ Some recent studies with data mainly from high-income countries (HICs) demonstrated that SGA children are at a greater risk of experiencing behavioural and cognitive difficulties such as lack of attention span altering academic performance and peer relations as compared with their AGA counterparts.^15^ One prospective study, which has followed children up to 30 years of age, reported 33% higher odds of lower educational achievement among SGA children as compared with AGA peers, indicating the significant impact of being SGA on the adult life course.^16^ Presently, there is paucity of literature from LMICs that demonstrate the neurodevelopmental impacts of SGA.^12^ Therefore, the objective of this study was to assess differences in neurodevelopmental assessment of SGA and AGA children <5 years of age residing in a periurban community in Pakistan.

## Methods

This prospective cohort study conducted from May to October 2021, followed children from a pilot cohort study (conducted in 2018) of women who had fetal Doppler ultrasound in order to develop a pregnancy risk stratification model using machine learning to predict adverse perinatal outcomes.^17^ The study was conducted in Ibrahim Hyderi, a periurban fishing community in the southeast of Karachi, Pakistan. As part of the pilot Doppler study, pregnant women were enrolled in the second trimester, had fetal Doppler scans and were followed until 1 week after delivery for neonatal outcomes. Children were labelled SGA (ie, birth weight ≤10th centile for gestational age) based on the INTERGROWTH standards.^18^ This project led to the creation of a community-based cohort of 650 children with 18% (n=119) SGAs.

For the current study, the sample size required to assess the neurodevelopmental changes was based on similar work conducted on preterm children in Uganda.^19^ This study reported that 20.4% children born preterm were at risk of neurodevelopmental delays as compared with 7.5% term children.^19^ Using these assumptions, 80% power and 5% level of significance, a sample size of 90 children each was obtained in the SGA and AGA groups.

Due to the ongoing maternal and child surveillance in these areas, all SGA children (n=119), from the pilot Doppler study were approached to participate. A list of 531 AGA children was available from the same pilot cohort from which every second child was approached until the required number of AGA sample was achieved. Study details were provided to the family, and written informed consent was obtained from parents before enrollment. Once consented, the children and the mother/guardian were brought to the primary healthcare centre located within the community for detailed history regarding the child’s history, anthropometry and neurodevelopmental assessment.

### Neurodevelopment assessments

The enrolled children were assessed for neurodevelopment using the Malawi Developmental Assessment Tool (MDAT). The MDAT is a culturally appropriate tool, has good psychometric properties and has proven to be reliable (kappa>0.75), sensitive (97%) and specific (82%) in identifying developmental delays in children up to the age of 6 years.^20 21^ MDAT has been translated, adapted and validated in many LMICs, including Pakistan.^19 22^ This tool assesses four domains including fine motor, gross motor, social and language (with cognitive items mainly included in fine motor and language domains) and is based on caregiver reporting, passive observation and direct administration.^23^ This comprehensive neurodevelopmental assessment tool is easily accessible, requires minimal training, takes no longer than 45 min to administer and is cost-effective in LMICs.^24^

The MDAT assessors were trained for 3 days and performed hands-on practice on children of a similar age under the supervision of an ECD expert. Quality checks through video recordings and periodic checking of the data was conducted by a trained supervisor. The child’s age was calculated before starting the assessment by subtracting the date of birth from the date of assessment to find the starting point for each domain, adjusted for gestational age at delivery.

The MDAT data were recoded using Mariza coding from the Shiny application dictionary.^25^ The child’s age was converted into years, and the recoded file was imported to the Shiny application to calculate the z-scores. The z-scores were categorised into binary category of pass and fail using the cut-off >−2 z-scores and ≤−2 z-scores, respectively, for SGA and AGA. Data were presented as mean±SD, median (IQR) or percentages as appropriate. Neurodevelopmental scores were compared among the SGA and AGA groups. Multivariable linear regression was used to compare the absolute scores, while multivariable logistic regression was used to compare the z-scores. Univariate analysis was conducted to compute crude regression coefficients with 95% CIs, where a p value of <0.25 made the variable eligible for inclusion in the multivariable analysis. A stepwise approach was used during multivariable analysis while adjusting for confounders such as woman’s and husband’s education, maternal midupper arm circumference (MUAC), parity, gestational age, tobacco use, child’s current age and presence of stunting. A p value of <0.05 was considered significant. Data were analysed using Stata (V.14.2, Statacorp).

## Results

The mean age of the mother was 26.7±5.5 years, with 56% (n=101) of them having no formal education. Seventy-two per cent of pregnant women (n=130) were multiparous, and 23% (n=42) were malnourished with a MUAC <23 cm. Thirty-five per cent (n=64) women reported pregnancy complications (refer to footnote in table 1). The mean age of enrolled children was 2.7±0.2 years. While comparing the nutritional status of children, SGA children had a higher frequency of stunting (54% vs 43%), wasting (14.4% vs 5.6%) and underweight (54% vs 31%) as compared with the AGA children (table 1).

**Table 1:** Baseline characteristics of parents and children

Maternal characteristics	Total (n=180) n (%)	SGA (n=90) n (%)	AGA (n=90) n (%)	P value
Age of mother (years), mean±SD	26.7 (5.5)	26.4 (6.1)	26.9 (4.9)	0.46
Education of mother				0.92
No formal education	101 (56.1)	51 (56.7)	50 (55.6)	
Primary	32 (17.8)	15 (16.7)	17 (18.9)	
Secondary	47 (26.1)	24 (26.7)	23 (25.5)	
Occupation of mother				0.65
Employed	5 (2.8)	3 (3.3)	2 (2.2)	
Unemployed	175 (97.2)	87 (96.7)	88 (97.8)	
Ever sniffed/chewed tobacco				0.63
Currently sniffing/chewing	57 (31.7)	27 (30)	30 (33.3)	
Never	123 (68.3)	63 (70)	60 (66.6)	
Ever chewed betel nut				0.18
Currently sniffing/chewing	77 (42.8)	34 (38)	43 (48)	
Never	103 (57.2)	56 (62)	47 (52)	
Parity, median (range)	2 (1–16)	2 (1–7)	3 (1–16)	<0.001
Parity				0.008
Primary parous	50 (27.8)	33 (37)	17 (19)	
Multiparous	130 (72.2)	57 (63)	73 (81)	
Antenatal care from a skilled care provider				0.41
Yes	129 (71.7)	67 (74.4)	62 (68.9)	
No	51 (28.3)	23 (25.6)	28 (31.1)	
MUAC				0.48
Normal (≥23)	138 (76.7)	67 (74.4)	71 (78.9)	
Malnutrition (<23)	42 (23.3)	23 (25.6)	19 (21.1)	
Anaemia status (defined as Hb <11 g/dL)				0.22
No	28 (15.6)	11 (12.2)	17 (18.9)	
Yes	152 (84.5)	79 (87.8)	73 (81.2)	
Pregnancy complications*				0.12
Yes	64 (35.6)	37 (41)	27 (30)	
No	116 (64.4)	53 (59)	63 (70)	
Delivery assisted by				0.27
Skilled birth attendant	142 (78.9)	74 (82.2)	68 (75.5)	
Unskilled birth attendant	38 (21.1)	16 (17.8)	22 (24.4)	
Mode of delivery				0.84
Spontaneous vaginal delivery	151 (83.9)	76 (84.4)	75 (83.3)	
Caesarean section	29 (16.1)	14 (15.6)	15 (16.7)	
Place of birth of child				0.17
Facility	136 (75.6)	72 (80)	64 (71)	
Home	44 (24.4)	18 (20)	26 (29)	
Paternal characteristics				
Education of father				0.94
No formal education	105 (58)	52 (57.8)	53 (58.9)	
Primary	19 (11)	9 (10)	10 (11.1)	
Secondary	56 (31)	29 (32.3)	27 (30)	
Occupation of father				0.25
Employed	159 (88.3)	77 (85.5)	82 (91)	
Unemployed	21 (11.7)	13 (14.4)	8 (8.9)	
**Child characteristics**				
Gender				0.76
Male	102 (56.7)	50 (55.6)	52 (57.8)	
Female	78 (43.3)	40 (44.4)	38 (42.2)	
Birth weight (kg), mean±SD	2.7 (0.5)	2.4 (0.4)	3.0 (0.5)	<0.001
Gestational age				0.004
Preterm (<37 weeks)	33 (18.3)	9 (10)	24 (27)	
Term (≥37 weeks)	147 (81.7)	81 (90)	66 (73)	
Current age (years), mean±SD	2.7 (0.2)	2.7 (0.2)	2.7 (0.2)	0.44
Current weight (kg), mean±SD	10.9 (1.4)	10.45 (1.2)	11.5 (1.4)	<0.001
Malnutrition status				
Stunting	88 (48.9)	49 (54)	39 (43)	0.14
Wasting	18 (10.0)	13 (14.4)	5 (5.6)	0.047
Underweight	77 (42.8)	49 (54)	28 (31)	0.002

*Pregnancy complications include the presence of any one of the conditions: vaginal bleeding, pregnancy-induced hypertension, diabetes, convulsions and overnight stay in facility due to fever.

AGA, appropriate for gestational age; MAUC, maternal midupper arm circumference; SGA, small for gestational age.

**Figure 1:**
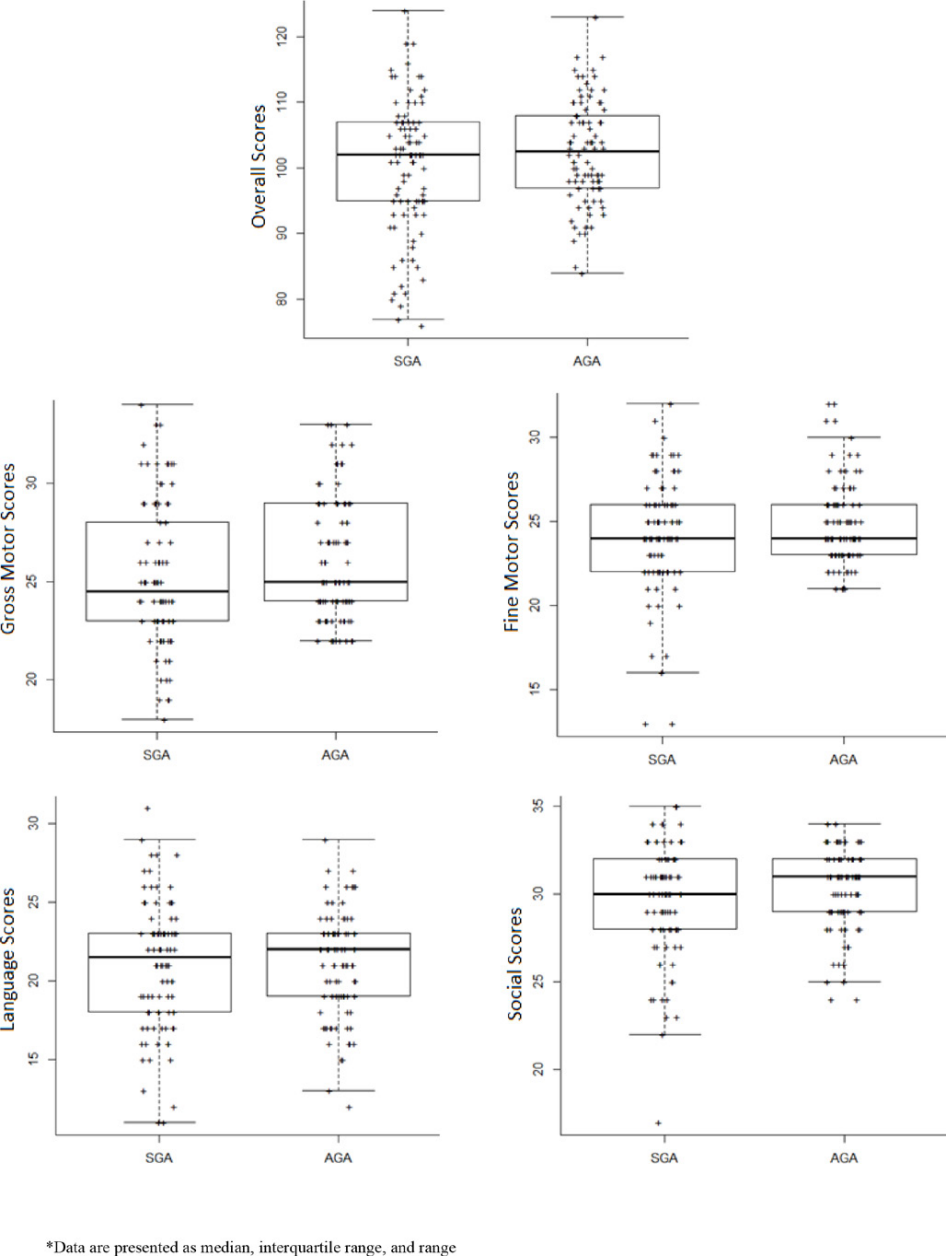
Distribution of scores for overall and in each domain of the MDAT among SGA and AGA. Data are presented as median, IQR and range. AGA, appropriate for gestational age; SGA, small for gestational age.

Figure 1 presents the distribution of overall and domain scores assessed in the two groups using MDAT. It can be seen that higher number of children in the SGA group had lower scores on the overall and all MDAT domains as compared with AGA children. The difference was more pronounced in the fine and gross motor domains.

A multivariable linear regression analysis showed that children born SGA had significantly lower scores on the fine motor domain (β: −0.98; 95% CI −1.90 to –0.06) as compared with the AGA children (table 2). The scores on the other domains, including gross motor, language, social as well as overall development were also lower among SGA children; however, none of these reached statistical significance.

**Table 2:** Multivariable linear regression of risk factors associated with neurodevelopment scores among SGA and AGA

	SGA (n=90)	AGA (n=90)	β (95% CI)
	Absolute scores	Absolute scores	
Overall scores	100.12	102.36	−2.42 (−5.14 to 0.29)
Gross motor scores	25.19	26.07	−0.86 (−1.89 to 0.18)
Fine motor scores	24.2	24.91	−0.98 (−1.90 to −0.06) *
Language scores	21.01	21.06	−0.24 (−1.39 to 0.90)
Social scores	29.72	30.32	−0.30 (−1.15 to 0.54)

*Significant finding.

†Adjusted for woman and her husband’s education, maternal MUAC, parity, sniffed tobacco, child’s age, stunting and gestational age at delivery.

AGA, appropriate for gestational age; MUAC, maternal midupper arm circumference; SGA, small for gestational age.

While comparing the neurodevelopment z-scores among SGA and AGA children, a multivariable logistic regression analysis showed that SGA children had significantly higher odds of having ≤−2 z-scores on overall MDAT (OR: 3.78; 95% CI 1.20 to 11.89) as compared with the AGA children. Although there were greater number of SGA children with −2 z-scores on all individual and overall domains, only the overall scores reached statistical significance. Statistical tests were not applied on the other three domains as a small number of AGA children fell in the ≤−2 z-scores category (table 3).

**Table 3:** Logistic regression of risk factors associated with neurodevelopment z-scores among SGA and AGA

	SGA (n=90)	AGA (n=90)	Or (95% CI)
	≤−2 z-scores (n)	≤−2 z-scores (n)	
Overall scores	14	6	3.78 (1.20 to 11.89)*
Gross motor scores	9	1	Not done
Fine motor scores	7	0	Not done
Language scores	16	11	1.83 (0.71 to 4.68)
Social scores	1	0	Not done

*Significant finding.

†Adjusted for woman and her husband’s education, maternal MUAC, parity, sniffed tobacco, child’s age, stunting and gestational age at delivery.

AGA, appropriate for gestational age; MUAC, maternal midupper arm circumference; SGA, small for gestational age.

**Figure 2:**
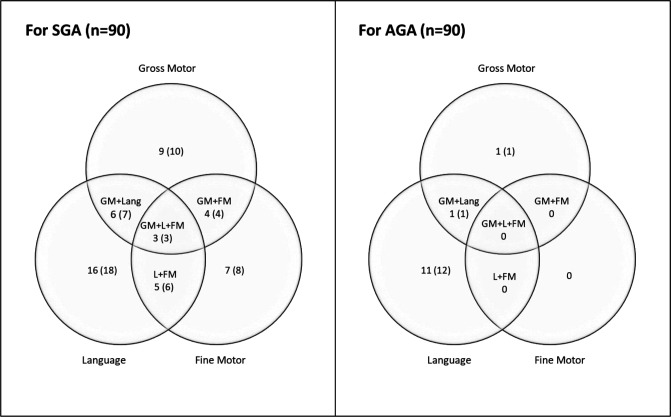
Distribution of SGA and AGA children who had ≤−2 z-ccores on gross motor, fine motor and language domains of MDAT N (%). AGA, appropriate for gestational age; FM, fine motor; GM, gross motor; L, language; SGA, small for gestational age.

Figure 2 depicts the distribution of SGA and AGA children whose z-scores were ≤−2 SD on each of the domains. Eighteen per cent (n=16) of the SGA children were delayed in language, 10% (n=9) in gross motor and 8% (n=7) in fine motor domain. The overlap between the three domains also demonstrated that more SGA children were delayed in two domains as compared with AGA children. There were three children from the SGA group who were delayed on gross and fine motor as well as language domain. Only one SGA child was delayed in social domain.

## Discussion

This study aimed to assess neurodevelopmental outcomes in children born SGA from a community-based cohort in Pakistan. The findings demonstrated that children born SGA had three times higher odds of ≤−2 z-scores on overall MDAT and significantly lower fine motor scores on MDAT assessment as compared with AGA children. In addition, overall developmental scores and other domain scores (gross motor, language and social) were all lower in SGA children; however, the findings were not statistically significant.

Children born SGA are at a greater risk of developing neurodevelopmental issues, including lower intelligence, psychomotor delays, low social competence and cognitive impairment, which are consistent with the findings of the present study.^26^ Therefore, early identification of these children for neurodevelopmental manifestations is essential for timely intervention. The neurodevelopment outcomes of SGA versus AGA children have been studied using various assessment tools with multiple domains.^21^ The selection of the assessment tool depends on the validity, reliability and cultural adaptability of the tool as well as ease to administer and training requirements of the accessor.^27^ The MDAT assesses children’s developmental milestones in a contextually relevant manner in LMICs.^23^ For example, the fine motor domain of MDAT uses items such as making a tower using blocks, putting pegs into the board, etc, which are appropriate to our cultural norms.^23^ However, other tools that are designed and validated in HICs use gestures and materials that are not culturally specific and hence may lead to misinterpretation on the domain.^23 28^

The existence of an increased risk of neurodevelopmental delay among SGA children in HICs has been found in previous literature, however, the evidence is contradictory. Savchev *et al* 29 assessed neurodevelopment outcomes among 2-year-old SGA children using Bayley-lll scale, which found significantly lower scores on language and adaptive domains. A Spanish study reported significantly lower neurodevelopmental centiles for problem-solving and personal-special domains among SGA children aged 2 years using Ages & Stages Questionnaire.^30^ In contrast, studies in the same age group from Ireland and Germany found no significant differences between SGA and AGA for the neurodevelopmental outcome using Bayley-lll and Griffiths Development Scale, respectively.^31 32^ These findings may be inconsistent due to different neurodevelopment assessment tools that were not validated and culturally representative of the population.^33 34^ Our study found an increased number of SGA children with overall neurodevelopmental deficits, mainly due to the fine motor domain. Although the difference was small, but it is clinically important as even subtle changes on the fine motor domain are closely linked to the cognitive abilities of children (creating patterns of blocks, filling pegboards, drawing shapes, etc).^35^ These findings are consistent with another study that demonstrated significantly impaired fine motor scores in SGA children at school age (mean age 8.6 years).^36^

Early fine motor skills are known to correlate with childrens’ current and future school performances and, at the age of 2 to 3 years, are particularly linked to cognition. Studies have demonstrated that children with strong fine motor skills perform better at preprimary school and have enhanced skills over time, especially in mathematics, whereas those with compromised skills had learning difficulties and required educational support.^36 37^ In contrast, children with a high composite score for fine and gross motor skills at preschool had significantly higher grades in primary school.^38^ MDAT has also demonstrated to serve as a screening tool to identify early developmental delays that are closely linked to children’s future cognitive functions.^39^ Boivin *et al* 39 have reported a significant correlation between MDAT and Kaufman Assessment Battery for Children (KABC-II), demonstrating the child’s early-age cognitive abilities with educational performance. These findings suggest that the fine motor changes observed in these studies may have implications on childrens’ school performance later in life. Furthermore, our results also found that a higher number of SGA children had lower z-score in any one or two domains on MDAT as compared with AGA children. Due to the lack of power on individual domains, the differences in the scores between the two groups are not statistically significant. However, these maybe clinically important as this depicts the possible burden of neurodevelopmental issues among SGAs and may provide grounds for screening for further referral and management of these children.

This study has several strengths and limitations. First, we used MDAT, which is explicitly designed for LMICs and validated in similar urban areas, including Pakistan.^22 23^ We had a uniquely placed cohort of children whose mothers were part of a pilot Doppler cohort study with accurate information on pregnancy-related factors, gestational age at delivery and neonatal data, including birth weight. One limitation of our study is the lack of power on individual domains of the MDAT tool. Also, the assessors were not blinded to the SGA and AGA status; thus, observer bias may be present. Furthermore, we performed the MDAT assessment at one single point in time and have not followed up with these children to assess neurodevelopmental trajectories. We also did not explore the aetiology of SGA in our cohort, the pathophysiology of which may be associated with a different phenotype of neurodevelopmental outcome.

This observational study reported significant differences in fine motor skills in SGA as compared with AGA children from a periurban community in Karachi, Pakistan. Further research is needed to design follow-up studies to assess the impact of these findings on child’s school performance. It would also be important to correlate these findings with structural brain changes using low-cost, non-invasive, neuroimaging techniques. Such robust assessments would help with prompting early assessment and timely intervention of neurodevelopmental delays in SGA children, thus helping children reach their maximum potential and achieve the ECD objectives of Sustainable Development Goals.

## Data Availability

Data are available on reasonable request. Data are available on reasonable request by contacting the corresponding author and following acceptance by the contributing institution.
